# Nutritional scores predict the prognosis of patients with pulmonary tuberculosis

**DOI:** 10.3389/fnut.2024.1454207

**Published:** 2024-12-13

**Authors:** Junyi Tan, Xiaofeng Shi, Yinhuan Pi, Mingque Xiang, Tianju Li

**Affiliations:** ^1^Department of Infectious Diseases, The Second Affiliated Hospital of Chongqing Medical University, Chongqing, China; ^2^Department of Infectious Diseases, The Ninth People’s Hospital of Chongqing, Chongqing, China

**Keywords:** pulmonary tuberculosis, malnutrition, controlling nutritional status, prognostic nutritional index, Naples prognostic score, body mass index, nutrition risk screening-2002, prognosis

## Abstract

**Background:**

Although malnutrition is associated with poor prognosis in Pulmonary Tuberculosis (PTB) patients, no nutrition-based prediction model has been established for PTB. Herein, we explored the clinical utility of common nutrition scores in predicting the prognosis of PTB patients.

**Methods:**

We retrospectively collected clinical baseline data from 167 patients with secondary PTB who had not previously received anti-TB treatment. Subsequently, we determined the CONUT score, PNI index, and NPS score and evaluated the treatment efficacy using changes in lung lesions revealed by the chest CT scan. The Area Under the Receiver Operating Characteristic (AUROC) curve was used to quantify the predictive values of CONUT, PNI, and NPS scores for anti-TB efficacy in new-onset PTB patients, and the critical CONUT, PNI, and NPS values were determined using the Youden Index. We also performed univariate and multivariate analyses of prognostic factors in PTB patients to determine the nutrition scores and other clinical factors associated with the prognosis of patients with the new-onset PTB.

**Results:**

The Youden Index revealed that the critical CONUT score value for patients with PTB was 4.5, with a sensitivity of 72.2% and specificity of 96.6%. In contrast, the critical cut-off values of the PNI index and the NPS score were 39.825 and 3.5, respectively. Univariate analysis of the predictors of poor prognosis in PTB patients showed that patients with diabetes, COPD, pneumonia, and hypoproteinemia (which were risk factors) had a poor prognosis (*p* < 0.05). Multivariate analysis revealed that the CONUT score, PNI, NPS, and NRS-2002 were independent predictors of unfavorable PTB outcomes, with adjusted ORs of 60.419 (95%CI: 16.186–225.524, *p* < 0.0001), 23.667 (95% CI: 9.317–60.115, *p* < 0.0001), 8.512 (95% CI: 3.762–19.257, *p* < 0.0001), 0.612 (95% CI: 4.961–39.161, *p* < 0.0001), respectively. The Area Under the Curve (AUC) of the CONUT score in predicting poor prognosis of PTB patients was 0.885 (95% CI:0.830–0.940, *p* < 0.0001), which is comparable to that of the PNI index (0.862, 95% CI: 0.805–0.920, *p* < 0.0001), but higher than that of NPS (0.774, 95% CI: 0.702–0.846, *p* < 0.0001), BMI (0.627, 95% CI: 0.541–0.717, *p* < 0.0001), and NRS-2002 (0.763, 95% CI: 0.688–0.838, *p* < 0.0001). We discovered that older patients (*p* < 0.0001), male participants (*p* < 0.05), and patients with Diabetes Mellitus (DM) (*p* < 0.0001) and Chronic Obstructive Pulmonary Disease (COPD) (*p* < 0.05) were more likely to have a high CONUT score.

**Conclusion:**

The poor prognosis of PTB patients was related to a high CONUT score, low PNI index, and high NPS score, of which the specificity and sensitivity of the CONUT score were higher than those of the PNI index and the NPS score.

## 1 Introduction

Tuberculosis (TB), a chronic disease caused by *Mycobacterium tuberculosis* (MTB), will continue to be a major global public health problem. In 2022, TB incidence and mortality were reported to be 10.6 million and 1.3 million people, respectively, with a significantly higher prevalence in developing countries than in developed economies (1). The lung is the body organ most susceptible to MTB, with reported lung involvement rates in subjects with active TB ranging between 79 and 87% (2, 3).

In all pathogen infections, a complex interplay between host response and microbial virulence, which regulates the overall metabolic response as well as the extent of tissue damage, prevails. According to research, TB leads to malnutrition, and malnourished patients are highly susceptible to TB (4). Malnutrition is considered an independent risk factor for TB recurrence (5) as well as an important and potentially reversible risk factor for treatment failure (6). Malnutrition can be assessed in various ways, and although BMI and NRS-2002 are readily available and simple to use; hence, they are commonly employed, they mostly focus on macro factors such as patient disease status and body weight (7). According to previous research, albumin levels, lymphocyte count, cholesterol content, and other laboratory results are related to the prognosis of PTB patients (8). Therefore, based on nutritional assessment, more reliable combined scoring systems such as the Controlling Nutritional Status (CONUT) score, the Prognostic Nutritional Index (PNI), and the Naples Prognostic Score (NPS) were developed to accurately predict patient prognoses. Herein, we hope to verify the applicability of these screening systems in patients who are most likely to benefit from nutritional interventions and better inform anti-TB therapy.

The CONUT score, an objective and easy-to-apply biomarker for predicting the prognosis of cancer patients (9, 10), encompasses serum albumin levels, cholesterol content, and lymphocyte counts (11) (Table 1). On the other hand, the PNI score is determined based on the following formula: (10 × albumin level [g/dL]) + (0.005 × lymphocyte count [number/mm3]) (12). It was originally designed to determine the risk of postoperative complications following Gastrointestinal (GI) surgery. Simple calculations employing experimentally determined formulas can quantify the nutritional status associated with patient outcomes. Finally, Galizia et al. developed a new research method, the NPS Score (Table 2), a scoring system comprising serum albumin content, Total Cholesterol (TC) level, Neutrophyl-to-Lymphocyte Ratio (NLR), and the Lymphocyte-to-Monocyte Ratio (LMR), that can comprehensively reflect patients’ immune and nutritional status (13). These three systems have been demonstrated to predict the prognosis of patients with Non-Small Cell Lung Cancer (NSCLC), breast cancer, and GI tumors (14–16). However, the correlation of the CONUT score, the PNI index, and the NPS score with outcomes in TB patients has not been reported. As a result, this study aims to explore the predictive value of the above-mentioned screening systems in the prognosis of TB patients, and to compare their predictive ability.

**Table 1:** Scoring methods for the CONUT score.

Variables	Degree of nutritional status			
	Normal	Mild	Moderate	Serve
Serum albumin concentration (g/dL)	≥3.50	3.0–3.49	2.50–2.99	<2.5
Score	0	2	4	6
Total lymphocyte count (/mm3)	≥1600	1200–1599	800–1199	<800
Score	0	1	2	3
Total cholesterol concentration (mg/dL)	≥180	140–179	100–139	<100
Score	0	1	2	3
Total score	0–1	2–4	5–8	9–12

CONUT denotes controlling nutritional status.

**Table 2:** Scoring methods for the NPS score.

Variables	Degree of nutritional status	
	Score	Score
Plasma albumin concentration (g/dL)	≥4.0	<4.0
Score	0	1
Total cholesterol concentration (mg/dL)	>180	≤180
Score	0	1
N/L	≤2.96	>2.96
Score	0	1
L/M	>4.44	≤4.44
Score	0	1
Total score	>0	>2
	Mild malnutrition risk	Severe malnutrition risk

NPS denotes Naples prognostic score.

## 2 Methods

### 2.1 Patients

We retrospectively collected clinical data on patients with new-onset secondary PTB who hospitalized in TB-designated hospitals of Beibei district (the Nine People’s Hospital of Chongqing) between January 2021 and December 2021.

The inclusion criteria were as follows: (1) Patients clearly diagnosed with secondary PTB per the diagnostic criteria for secondary PTB (WS288-2017) (17); (2) Patients positive for MTBC (*Mycobacterium tuberculosis* Complex) who returned negative results for RIF resistance per the Xpert MTB/RIF (Rifampicin) test; (3) Patients administered with first-line antituberculosis drugs such as rifampicin (R), isoniazid (H), ethambutol (E), and pyrazinamide (Z), and adopted specific treatment courses and plans based on the patient’s weight and comorbidities; and (4) Patients who underwent chest CT scan again at the end of 6 months of anti-TB treatment for evaluating PTB lesions.

The exclusion criteria were as follows: (1) Patients not on drugs or any regular follow-up assessments; (2) Patients who have previously received anti-TB treatment or those with missing data on their TB condition pre-treatment. (3) Patients on long-term use of Glucocorticoids (GCs) or drugs affecting albumin levels and lymphocyte count.

### 2.2 Data collection

We obtained clinical data on PTB patients before anti-TB treatment from the hospital’s Information Management System (IMS) and used it as the baseline. Specifically, we collected data on general demographics (age, gender, occupation, days of hospital stay, and so on), drug and alcohol use history (such as smoking and drinking), comorbidities [such as hypertension, Diabetes Mellitus (DM), renal insufficiency, hyperlipidemia, Chronic Obstructive Pulmonary Disease (COPD), and Acquired Immunodeficiency Syndrome (AIDS)], laboratory tests (such as blood routine tests, liver function tests, kidney function tests, blood lipids, sputum culture, sputum smear, chest CT, and so on) and complications, (such as respiratory failure, pulmonary infection, and so on).

The efficacy of anti-TB drug treatment was assessed through chest Computed Tomography (CT) scans to observe radiological features per the national guidelines.

(1) Infiltrated lesions
  - Obvious absorption: Lesion absorption ≥1/2 of the original lesion.
  - Absorption: Lesion absorption <1/2 of the original lesion.
  - Unchanged: No significant change in the lesion.
  - Worsening: Lesion expansion or dissemination.
(2) Cavities
  - Closure: Obstructive closure and scar closure or disappearance.
  - Reduction: Cavity reduction ≥ ½ of the original cavity diameter.
  - Unchanged: Cavity reduction or increase < ½ of the original cavity diameter.
  - Increase: Cavity increase ≥ ½ of the original cavity diameter.

This study was approved by the Ethical Review Committee of the Ninth People’s Hospital of Chongqing for the use of clinical data, and was conducted per the 1964 Declaration of Helsinki. All patients gave informed consent before participating herein.

All statistical analyses were performed using SPSS v25.0. Continuous variables were expressed as Mean ± Standard Deviation (SD) and compared using two independent sample *t*-tests or Mann–Whitney *U* tests. On the other hand, categorical variables were expressed as counts (N) or percentages (%) and compared using the chi-square test or Fisher’s exact test. The Area under the Receiver Operating Characteristic (AUROC) curve of the six-month anti-TB treatment efficacy was used to quantify the predictive value of CONUT, PNI, NPS, BMI and NRS-2002 for the anti-TB efficacy in treatment-naive new onset PTB patients. The maximum Youden Index value was used as the best cut-off value to classify patients into two groups. Spearman’s correlation was used to evaluate the correlation of the key five parameters (CONUT, PNI, NPS, BMI, and NRS-2002) with prognosis, as well as among the five individual measures. Univariate and multivariate Logistic regression analyses were performed to determine the independent predictors of anti-TB treatment efficacy, and the Odds Ratio (OR) and 95% Confidence Interval (CI) were calculated. Results with *p* < 0.05 were considered statistically significant.

## 3 Results

### 3.1 Clinical features

Table 3 summarizes the clinical characteristics of 167 PTB patients before anti-TB treatment. Age ranged between 14 and 85 years, with an average of 53 years. Days of hospital stay ranged between 3 and 122 days, with an average of 23 days. Among the patients enrolled, 88 (52.69%), 37 (22.16%), and 4 (2.40%) had a history of smoking, drinking, and dust work, respectively. Additionally, among the patients, 29 (17.37%), 29 (17.37%), 3 (1.80%), 8 (4.79%), 16 (9.58%), and 11 (6.59%) had hypertension, diabetes, renal insufficiency, hyperlipidemia, COPD, and AIDS, respectively. Major complications included pneumonia and hypoproteinemia in 10 (5.99%) and 11 (6.59%) patients, respectively. Furthermore, among the patients, 42 (25.15%) were underweight (BMI < 18.5 kg/m2), 89 (53.29%) had NRS-2002 scores ≥3 and were at risk of malnutrition, 60 (35.93%) had CONUT scores >4.5, 69 (41.32%) had PNI ≤39.825, and 81 (48.50%) had NPS scores >3.5.

**Table 3:** Characteristics of PTB patients.

Characteristics	Value or no. of patients	Characteristics	Value or no. of patients
Age (years)		AIDS	
Mean (SD)	53.12 (18.51)	Present	11 (6.59%)
Gender		Complications	
Male	125 (74.85%)	Pneumonia	
Hospital stay (days)		Present	10 (5.99%)
Mean (SD)	23.04 (17.67)	Hypoproteinemia	
Smoking status		Present	11 (6.59%)
Present	88 (52.69%)	Nutritional scores	
Drinking status		BMI	
Present	37 (22.16%)	<18.5 kg/m2	42 (25.15%)
Dust-exposed work		≥18.5 kg/m2	125 (74.85%)
Present	4 (2.40%)	NRS2002	
Basic diseases		≥3	89 (53.29%)
Hypertension		<3	78 (46.71%)
Present	29 (17.37%)	CONUT	
Diabetes mellitus		> 4.5	60 (35.93%)
Present	29 (17.37%)	≤ 4.5	107 (64.07%)
Renal insufficiency		PNI	
Present	3 (1.80%)	> 39.825	98 (58.68%)
Hyperlipidemia		≤ 39.825	69 (41.32%)
Present	8 (4.79%)	NPS	
COPD		> 3.5	81 (48.50%)
Present	16 (9.58%)	≤ 3.5	86 (51.50%)
≤ 3.5	86 (51.50%)		

PTB denotes Pulmonary Tuberculosis, COPD denotes Chronic Obstructive Pulmonary Disease; AIDS denotes Acquired Immunodeficiency Syndrome; BMI denotes Body Mass Index; NRS-2002 denotes Nutrition Risk Screening-2002; CONUT denotes Controlling Nutritional Status; PNI denotes Prognostic Nutrition Index; NPS denotes Naples Prognostic Score; and SD denotes Standard Deviation.

### 3.2 Univariate analysis of prognosis

Demographics, clinical features, and laboratory test results were subjected to univariate statistical analysis in relation to PTB prognosis (Table 4). Age (*p* < 0.0001) and days of hospital stay (*p* < 0.05) were found to be risk factors for the prognosis of PTB patients. Patients with DM (*p* < 0.05), COPD (*p* < 0.05), and pneumonia (*p* < 0.05) were more likely to have adverse outcomes. Among the laboratory test results, TC (*p* < 0.0001), albumin (*p* < 0.0001), N/L (*p* < 0.0001), M/L (*p* < 0.05), and P/L (*p* < 0.05) were significantly associated with an unfavorable outcome. On the other hand, nutritional assessment revealed that BMI (*p* < 0.05), NRS-2002 (*p* < 0.0001), CONUT (*p* < 0.0001), PNI (*p* < 0.0001), and NPS (*p* < 0.0001) may be prognostic risk factors. All results were statistically significant.

**Table 4:** Demographic and clinical parameters in the univariate analysis of participating patients by the six-month prognosis outcome.

Characteristic	Total patients (*N* = 167)	Favorable outcome (*N* = 88)	Unfavorable outcome (*N* = 79)	*p*-value
Age (years), mean (SD)	53.12 (18.51)	46.48 (18.05)	60.52 (16.14)	<0.0001
Gender (male), *n* (%)	125 (74.85)	63 (71.59)	62 (78.48)	0.307
Hospital stay (days), mean (SD)	23.04 (17.67)	19.40 (14.46)	27 (20.01)	0.008
Smoking, *n* (%)	88 (52.69)	45 (51.14)	43 (54.43)	0.670
Drinking, *n* (%)	37 (22.16)	20 (22.73)	17 (21.52)	0.851
Dust work history, *n* (%)	4 (2.40)	3 (3.41)	1 (1.27)	0.385
Basic diseases				
Hypertension, *n* (%)	29 (17.37)	14 (15.91)	15 (18.99)	0.600
Diabetes mellitus, *n* (%)	29 (17.37)	9 (10.23)	20 (25.32)	0.022
COPD, *n* (%)	16 (9.58)	4 (4.55)	12 (15.19)	0.027
AIDS, *n* (%)	11 (6.59)	8 (9.10)	3 (3.80)	0.182
Hyperlipidemia, *n* (%)	8 (4.79)	3 (3.40)	5 (6.33)	0.385
Renal insufficiency, *n* (%)	3 (1.80)	1 (1.13)	2 (2.53)	0.509
Complications				
Pneumonia, *n* (%)	10 (5.99)	1 (1.13)	9 (11.39)	0.024
Hypoproteinemia, *n* (%)	11 (6.59)	4 (4.54)	7 (8.86)	0.270
Laboratory feature				
Total cholesterol (mg/dL), mean (SD)	160.27 (44.42)	175.84 (41.57)	142.93 (41.16)	<0.0001
Albumin (g/dL), mean (SD)	3.60 (0.66)	3.96 (0.53)	3.19 (0.55)	<0.0001
Blood creatinine (umol/L), mean (SD)	61.36 (20.17)	60.18 (20.84)	62.67 (19.46)	0.427
N/L	6.93 (7.17)	4.50 (4.38)	9.63 (3.87)	<0.0001
M/L	0.54 (0.61)	0.43 (4.70)	0.66 (0.028)	0.008
P/L	305.35 (314.41)	227.84 (27.29)	391.69 (130.93)	0.003
BMI, *n* (%)				0.012
<18.5 kg/m2	42 (25.15)	15 (17.05)	27 (34.18)	
≥18.5 kg/m2	125 (74.85)	73 (82.95)	52 (65.82)	
NRS2002, *n* (%)				< 0.0001
≥3	89 (53.29)	25 (28.41)	64 (81.01)	
<3	78 (46.71)	63 (71.59)	15 (18.99)	
CONUT score, *n* (%)				< 0.0001
Low (≤ 4.5)	107 (64.07)	85 (96.59)	22 (27.85)	
High (> 4.5)	60 (35.93)	3 (3.41)	57 (72.15)	
PNI, *n* (%)				< 0.0001
Low (≤ 39.825)	69 (41.32)	9 (10.23)	60 (75.95)	
High (> 39.825)	98 (58.68)	79 (89.77)	19 (24.05)	
NPS, *n* (%)				< 0.0001
Low (≤ 3.5)	86 (51.50)	67 (76.14)	19 (24.05)	
High (> 3.5)	81 (48.50)	21 (23.86)	60 (75.95)	

CONUT denotes Controlling Nutritional Status; PNI denotes Prognostic Nutrition Index; NPS denotes Naples Prognostic Score; BMI denotes Body Mass Index; NRS-2002 denotes Nutrition Risk Screening-2002; AIDS denotes Acquired Immunodeficiency Syndrome; COPD denotes Chronic Obstructive Pulmonary Disease; and SD denotes Standard Deviation.

### 3.3 Multivariate analysis of prognosis

Table 5 summarizes our conclusions after subjecting the statistically significant indicators in the univariate analysis to multivariate analysis with CONUT, PNI, NPS, and NRS-2002. Age and pneumonia were independent risk factors for prognosis in models 1, 2, 3, and 4. Multivariate analysis results (along with the adjusted ORs) revealed that the CONUT score (OR: 60.419; 95% CI: 16.186–225.524; *p* < 0.0001), PNI (OR: 23.667; 95% CI: 9.317–60.115; *p* < 0.0001), NPS (OR: 8.512; 95% CI: 3.762–19.257; *p* < 0.0001), and NRS-2002 (OR: 0.612; 95% CI: 4.961–39.161; *p* < 0.0001) were independent predictors of unfavorable PTB outcomes.

**Table 5:** Multivariate analyses of the involvement of different variables in PTB patients with poor functional outcomes at 6 months.

		*β*	OR	95% CI	*p* value
Model 1	Age (years)	−0.038	0.962	0.935–0.991	0.011
	Hospital stay (days)	−0.009	0.991	0.960–1.024	0.596
	COPD	−0.616	0.760	0.010–28.261	0.760
	Diabetes mellitus	−1.325	0.266	0.064–1.112	0.070
	Pneumonia	4.173	64.901	0.643–6547.398	0.076
	BMI	−0.620	0.538	0.169–1.713	0.294
	CONUT	4.101	60.419	16.186–225.524	<0.0001
Model 2	Age (years)	−0.036	0.965	0.938–0.992	0.013
	Hospital stay (days)	−0.003	0.997	0.967–1.028	0.838
	COPD	−0.057	0.945	0.036–24.845	0.945
	Diabetes mellitus	−1.740	0.175	0.048–0.645	0.009
	Pneumonia	3.874	48.126	0.811–2856.210	0.063
	BMI	−0.487	0.615	0.202–1.880	0.394
	PNI	3.164	23.667	9.317–60.115	<0.0001
Model 3	Age (years)	−0.048	0.953	0.929–0.970	<0.0001
	Hospital stay (days)	−0.009	0.991	0.964–1.017	0.489
	COPD	−0.440	0.644	0.057–7.313	0.723
	Diabetes mellitus	−1.416	0.243	0.074–0.794	0.019
	Pneumonia	3.513	33.543	1.161–969.058	0.041
	BMI	−0.860	0.423	0.158–1.134	0.087
	NPS	2.141	8.512	3.762–19.257	<0.0001
Model 4	Age (years)	−0.041	0.960	0.936–0.984	0.001
	Hospital stay (days)	−0.013	0.987	0.961–1.014	0.338
	COPD	0.041	1.042	0.056–19.493	0.978
	Diabetes mellitus	−1.723	0.178	0.056–0.550	0.003
	Pneumonia	3.243	0.094	0.573–1145.346	0.094
	BMI	0.838	2.312	0.772–6.923	0.134
	NRS-2002	−0.491	0.612	4.961–39.161	<0.0001

CONUT denotes Controlling Nutritional Status; PNI denotes Prognostic Nutrition Index; NPS denotes Naples Prognostic Score; BMI denotes Body Mass Index; NRS-2002 denotes Nutrition Risk Screening-2002; CI denotes Confidence Interval; and OR denotes Odds Ratio.

### 3.4 Relationship between the CONUT score, PNI, NPS, BMI, and NRS-2002

**Figure 1:**
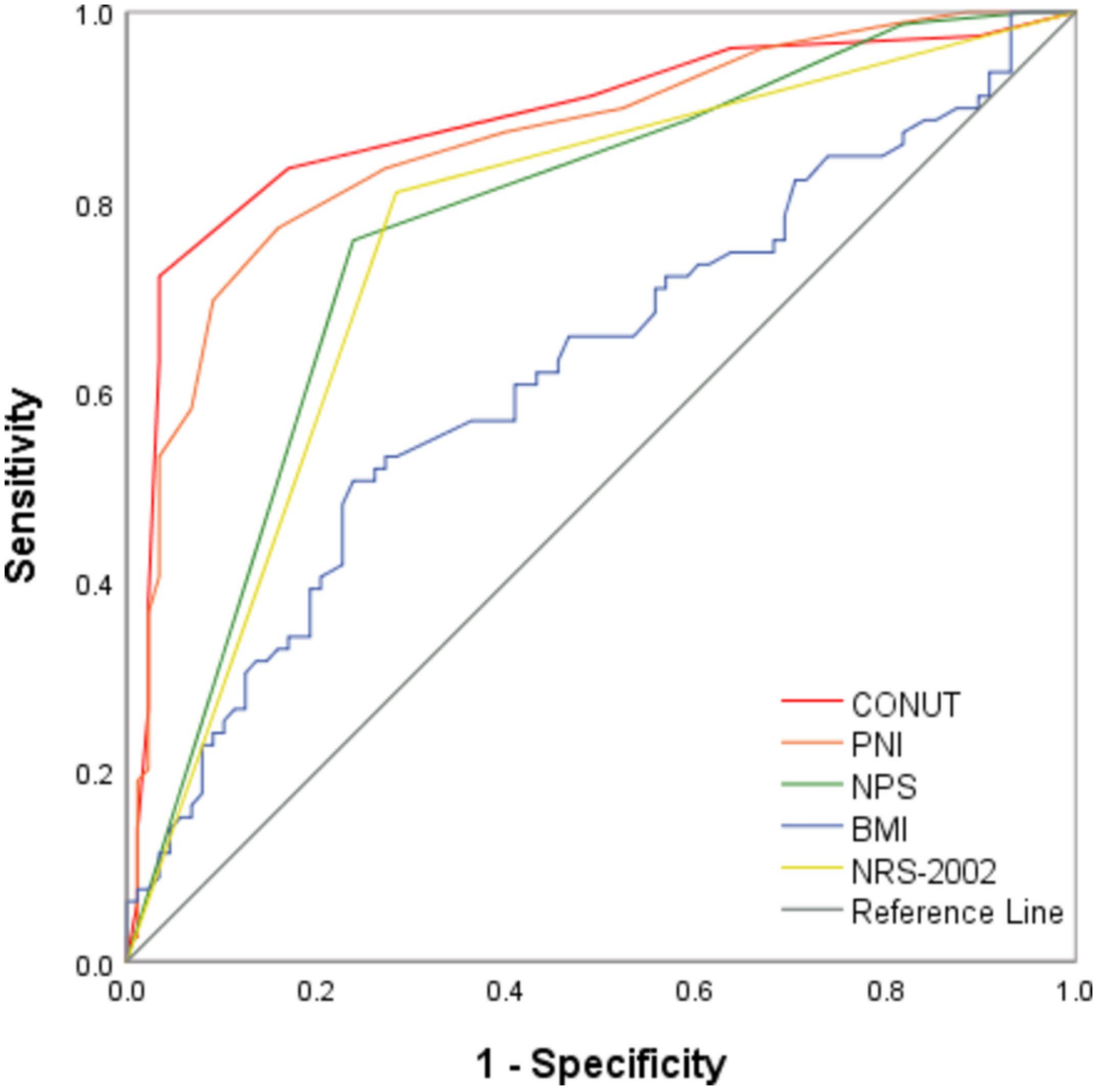
ROC analysis of CONUT, PNI, NPS, BMI, and NRS-2002 in predicting the functional outcome of PTB. CONUT denotes Controlling Nutritional Status; PNI denotes Prognostic Nutrition Index; NPS denotes Naples Prognostic Score; BMI denotes Body Mass Index; NRS-2002 denotes Nutrition Risk Screening 2002; and PTB denotes Pulmonary Tuberculosis.

Based on the Youden index, the cut-off values for the CONUT score, the PNI index, and the NPS score were determined to be 4.5 [most appropriate for sensitivity (72.2%) and specificity (96.6%)], 39.825 [most appropriate for sensitivity (89.8%) and specificity (75.9%)], and 3.5 [most appropriate for sensitivity (75.9%) and specificity (76.1%)], respectively. The ROC curve analysis results revealed that the CONUT score had the best diagnostic value with the AUC value of 0.885 (95% CI: 0.830–0.940, *p* < 0.0001), which was comparable to that of the PNI score (AUC: 0.862; 95% CI: 0.805–0.920; *p* < 0.0001) (Figure 1), but higher than that of the NPS score (AUC: 0.774, 95% CI: 0.702–0.846, *p* < 0.0001), BMI (AUC: 0.627; 95% CI: 0.541–0.717, *p* < 0.0001), and NRS-2002 (AUC: 0.763; 95% CI: 0.688–0.838; *p* < 0.0001).

Among the 167 patients, 88 had a favorable outcome, including 85 with a CONUT score < 4.5, 79 with a PNI index >39.825, 67 with an NPS score ≤ 3.5, 73 with a BMI ≥ 18.5 kg/m2, and 63 with an NRS-2002 score < 3. According to the Spearman’s correlation analysis results, CONUT, PNI, NPS, BMI, and NRS-2002 were correlated with prognosis, with the CONUT score (*r* = −0.672, *p* < 0.0001), NPS (*r* = −0.510, *p* < 0.0001), and NRS-2002 (*r* = −0.526, *p* < 0.0001) having a negative correlation with prognosis, and PNI (*r* = 0.636, *p* < 0.0001) and BMI (*r* = 0.219, *p* < 0.05) having a positive correlation with prognosis (Tables 6.1–6.4). Therefore, we concluded that the CONUT score was strongly associated with the prognosis of PTB patients.

**Table 6.1:** Relationship between PNI and CONUT.

		CONUT	
		Low	High
PNI	Low	11	58
	High	96	2

CONUT denotes Controlling Nutritional Status; PNI denotes Prognostic Nutrition Index.

**Table 6.2:** Relationship between NPS and CONUT.

		CONUT	
		Low	High
NPS	Low	79	7
	High	28	53

CONUT denotes Controlling Nutritional Status; NPS denotes Naples Prognostic Score.

**Table 6.3:** Relationship between CONUT and BMI.

		CONUT	
		Low	High
BMI	Low	21	21
	High	86	39

CONUT denotes Controlling Nutritional Status; BMI denotes Body Mass Index.

**Table 6.4:** Relationship between CONUT and NRS-2002.

		CONUT	
		Low	High
NRS-2002	Low	76	2
	High	31	58

CONUT denotes Controlling Nutritional Status; NRS-2002 denotes Nutrition Risk Screening 2002.

**Figure 2:**
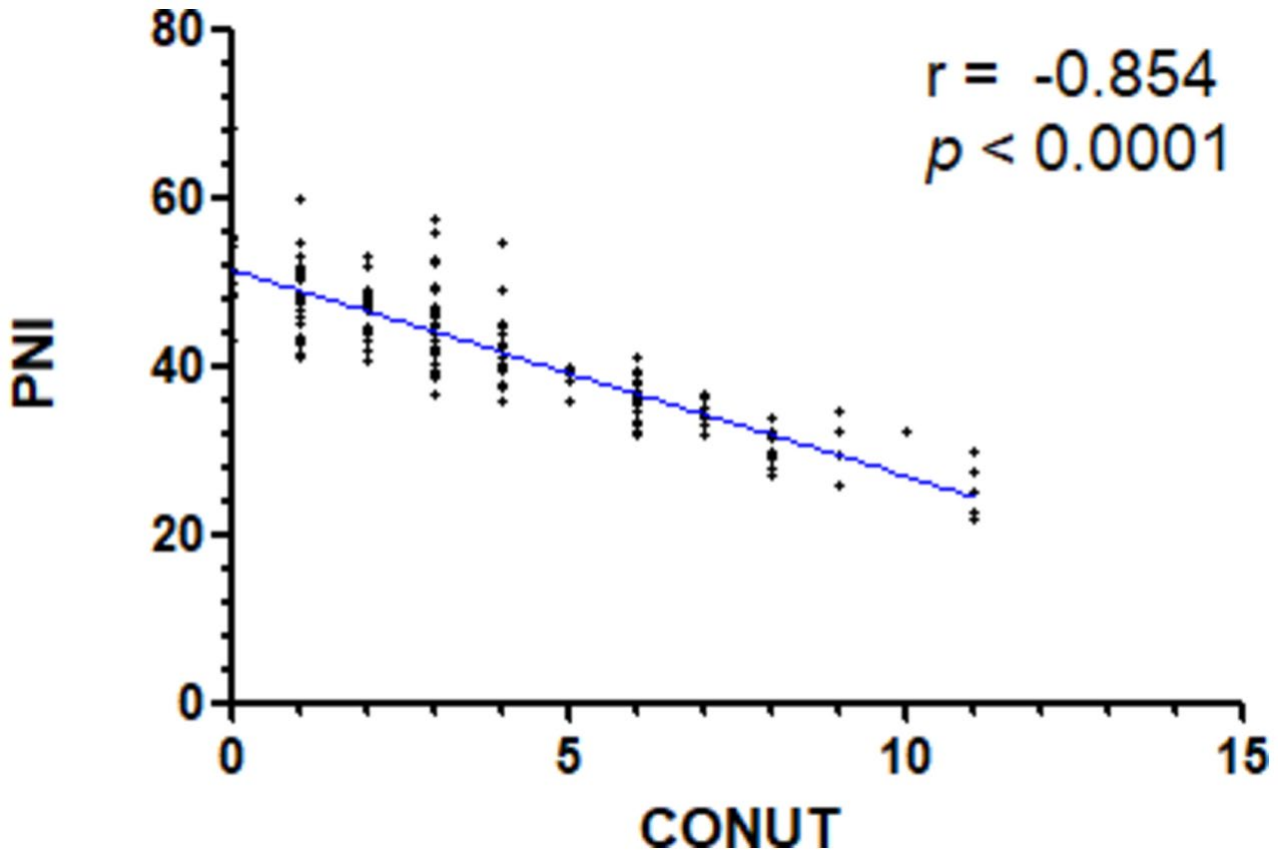
Spearman’s correlation analysis of the CONUT and PNI scores. CONUT denotes Controlling Nutritional Status; PNI denotes Prognostic Nutrition Index.

We also performed correlation analysis between the CONUT score and PNI (*r* = −0.854, *p* < 0.0001), NPS (*r* = 0.685, *p* < 0.0001), BMI (*r* = −0.295, *p* < 0.0001), and NRS-2002 (*r* = 0.647, *p* < 0.0001) in the context of nutritional parameters. A significant correlation was found between the PNI index and CONUT scores. The low CONUT and high PNI groups were largely overlapping (Figure 2).

### 3.5 Clinical features between HIV and non-HIV patients

Among the 167 participants in our study, 11 were co-infected with HIV. HIV infection is known to affect the immune and nutritional status of patients. Therefore, we conducted a statistical analysis of the clinical characteristics of HIV-positive and HIV-negative patients, as shown in Table 7. Low L counts and low protein concentrations are commonly detected in HIV patients, which is in agreement with findings from previous. However, there was no significant difference in CONUT scores between the two groups. Therefore, we believe that the CONUT score is well-suited for predicting the prognosis of pulmonary tuberculosis patients with varying nutritional conditions and comorbidities.

**Table 7:** Baseline clinical characteristics of patients with HIV patients and non-HIV patients.

Characteristic	HIV patients, (*N* = 11)	Non-HIV patients, (*N* = 156)	*p* value
Age (years), mean (SD)	52.91 (17.60)	53.13 (18.51)	0.774
Hospital stay (days), mean (SD)	29.73 (17.83)	22.52 (17.67)	0.042
WBC (/mm3), mean (SD)	5.82 (2.96)	7.60 (3.58)	0.064
L (/mm3), mean (SD)	0.83 (0.52)	1.34 (0.51)	0.044
N (/mm3), mean (SD)	4.50 (2.84)	5.82 (3.41)	0.189
M (/mm3), mean (SD)	0.38 (0.24)	0.51 (0.59)	0.500
Total cholesterol (mg/dL), mean (SD)	3.97 (1.13)	4.16 (1.15)	0.964
Albumin (g/dL), mean (SD)	32.03 (6.64)	36.23 (6.60)	0.016
CONUT (> 4.5), *n* (%)	7 (63.63)	53 (33.97)	0.058
PNI (≤ 39.825), *n* (%)	9 (81.82)	60 (38.46)	0.008
NPS (> 3.5), *n* (%)	5 (45.45)	76 (48.72)	1.000
BMI (< 18.5 kg/m2), *n* (%)	5 (45.45)	37 (23.72)	0.146
NRS2002 (≥ 3), *n* (%)	9 (81.82)	80 (51.28)	0.063

WBC, white blood cell; L, Lymphocyte; N, Neutrophils; M, Monocytes; PNI, Prognostic nutrition index; NPS, Naples prognostic score; BMI, Body Mass Index; NRS-2002, Nutrition risk screening-2002.

### 3.6 The difference in clinical features between the high CONUT and low CONUT scores

Based on the cut-off value of the CONUT score, patients were divided into two groups: Low CONUT score (≤4.5) and high CONUT score (>4.5). Patients in the high CONUT score group had a significantly higher average age (62 years) than those in the low CONUT score group (48 years) (*p* < 0.0001), and compared to their female counterparts, male patients were more likely to have a high CONUT score (*p* < 0.05) (Table 8). Compared to those in the low CONUT score group, patients in the high CONUT score group had a longer hospital stay (28 days on average, *p* < 0.0001) and a significantly higher prevalence of DM (*p* < 0.0001) and COPD (*p* < 0.05), which was more likely to be complicated with hyperlipidemia and hypoproteinemia (*p* < 0.05). Regarding the laboratory test results, compared to the low CONUT score group, the high CONUT score group had significantly lower albumin and cholesterol levels and significantly higher N/L, M/L, and P/L values. Regarding the nutritional assessment aspects, the low CONUT score group had a significantly better BMI than the high CONUT score group. On the other hand, the high CONUT score group showed significantly higher NRS-2002 scores than the low CONUT score group.

**Table 8:** Baseline clinical characteristics of patients with low CONUT and high CONUT scores.

Characteristic	Low-CONUT score (≤4.5), (*N* = 107)	High-CONUT score (>4.5), (*N* = 60)	*p*-value
Age (years), mean (SD)	47.88 (18.51)	62.47 (18.25)	< 0.0001
Gender (male), *n* (%)	71 (66.36)	54 (90.00)	< 0.05
Hospital stay (days), mean (SD)	20 (17.72)	28.33 (17.74)	< 0.05
Smoking, *n* (%)	53 (49.53)	35 (58.33)	0.333
Drinking, *n* (%)	21 (19.63)	16 (26.67)	0.334
Hypertension, *n* (%)	15 (14.02)	14 (23.33)	0.140
Diabetes mellitus, *n* (%)	7 (6.54)	22 (36.67)	< 0.0001
Dust work history, *n* (%)	2 (1.87)	2 (3.33)	0.619
COPD, *n* (%)	6 (5.60)	10 (16.67)	0.028
Pneumonia, *n* (%)	4 (3.74)	6 (10.00)	0.099
AIDS, *n* (%)	4 (3.74)	7 (11.67)	0.058
Hyperlipidemia, *n* (%)	2 (1.87)	6 (10.00)	0.026
Renal insufficiency, *n* (%)	1 (0.93)	2 (3.33)	0.293
Hypoproteinemia, *n* (%)	3 (2.80)	8 (13.33)	0.018
BMI (< 18.5 kg/m2), n (%)	21 (19.63)	21 (35.00)	0.040
NRS2002 (≥ 3), *n* (%)	31 (28.97)	58 (96.67)	<0.0001

BMI denotes Body Mass Index; NRS-2002 denotes Nutrition Risk Screening-2002; AIDS denotes Acquired Immunodeficiency Syndrome; COPD denotes Chronic Obstructive Pulmonary Disease; and SD denotes Standard Deviation.

## 4 Discussion

Malnutrition can weaken the immune system and increase the risk of infections by disrupting the production and secretion of cytokines (18). Severe malnutrition can impair various immune functions in the human body, including phagocytosis, cellular immunity, antibody concentration, and cytokine production, increasing susceptibility to MTB (19). In this, we explored the correlation between the nutritional status scores and outcomes following regular anti-TB treatment in patients diagnosed with new-onset PTB. We found that CONUT, PNI, NPS, and NRS-2002 are independent predictors of prognosis in PTB patients. Among them, CONUT score showed the strongest correlation with prognosis demonstrated the highest predictive value. Therefore, we suggest that high CONUT scores at the time of initial diagnosis are associated with a poor prognosis in anti-TB treatment. Elderly males, along with patients with diabetes and COPD, are more likely to present with higher CONUT scores.

Numerous investigations have demonstrated a two-way relationship between TB and malnutrition (20–22). On the one hand, TB patients have GI dysfunction and decreased appetite, leading to insufficient intake of nutrients, resulting in reduced anabolism (20). On the other hand, MTB uses body proteins for self-metabolism, resulting in physiological changes in individuals (22). The changes include low fever, night sweats, weight loss, and so on, leading to increased body catabolism, reduced fat storage, and loss of lean body tissue, further exposing patients to varying degrees of malnutrition. A nationwide multi-center, large-sample study reported that per the NRS-2002 classification, 55.86% of PTB patients are at risk of malnutrition, with 26.56% having a low BMI (<18.5 kg/m^2^) (23). These findings are consistent with our results which identified the rates of 53.29 and 25.15%, respectively. Previous studies have documented that cellular immunity is a critical component of the host’s defense mechanism against TB (24). Prolonged malnutrition could impair Cellular Immune Function (CMI), particularly the function of helper T lymphocyte CD4 cells, resulting in increased susceptibility to infections (25). Furthermore, the CD8 ratio of T lymphocytes decreases, which comprises the body’s normal immune function, thereby decreasing its ability to clear MTB, causing repeated TB procrastination, and further complicating PTB treatment (26). In this regard, malnutrition may affect TB prognosis. Consistent with most previous research findings (8, 27), we found that NRS-2002 and BMI were related to prognosis but with low correlation and predictive value. Other studies have shown that malnourished or immunocompromised patients do not experience significant weight loss or low BMI (28, 29). Therefore, besides the general screening factors, TB patients have their specific clinical characteristics, necessitating a more comprehensive and appropriate evaluation model specifically designed for TB.

Novel nutritional scores, e.g., comprising nutritional indicators (such as albumin and cholesterol) and immune indicators (such as lymphocytes) have been proposed. The PNI index includes albumin (nutritional indicator) and lymphocytes (immune indicator). The NPS score, on the other hand, comprises nutritional indicators, including lymphocytes, monocytes, and neutrophils. The combined use of individual nutritional indicators and immune indicators offers improved prognostic accuracy for acute conditions, such as severe traumatic brain injury (30). These indicators allow for quicker, more objective and precise prognostic assessments. However, further research is needed to explore their potential application in chronic diseases. Herein, we used a novel nutritional score for the prognostic assessment of PTB patients. Our findings revealed that CONUT, PNI, and NPS are all independent predictors of PTB prognosis, with the CONUT score having a higher specificity and sensitivity in predicting outcomes.

Serum albumin is widely considered a nutritional status indicator, and reduced serum albumin levels are often associated with inflammatory responses (31). Furthermore, albumin is one of the most sensitive nutritional status indicators in TB patients (32). A multitude of animal experiments have shown that hypoalbuminemia can alter the total number of T lymphocytes as well as the absolute and relative numbers of immune system cell subsets (33), reducing the host’s immunity to MTB (34). Low serum albumin levels, low total lymphocyte counts, and a low PNI index are highly associated with impaired immune function (8). Consistent with previous research, we found that albumin is positively associated with TB prognosis.

Cholesterol on the macrophage membrane is directly involved in MTB phagocytosis (35). The MTB infection dysregulates host lipid biosynthesis, uptake, and sequestration by altering the intracellular environment of macrophages (36). *In vivo* studies revealed have demonstrated that cholesterol is essential for MTB persistence during the latent phase of infection (37). Cholesterol is essential for efficient MTB internalization by macrophages (38) and is a vital energy source for MTB to maintain persistent infection (37). Lipid metabolism, on the other hand, is critical for TB virulence. Epidemiological studies have revealed a link between patients’ cholesterol levels and TB prognosis. Higher serum cholesterol levels have been shown to reduce TB bacterial load in sputum, increase the rate of sputum culture sterilization, and accelerate the conversion of sputum to negative, suggesting that elevated cholesterol levels are associated with faster bacterial clearance. These biomarkers, being low-cost and widely accessible, are useful for identifying patients with poor prognoses who may benefit from early nutritional interventions. Further prospective studies are needed to validate these findings (39). Similarly, our findings revealed a strong correlation between cholesterol and TB treatment efficacy, with high pre-treatment cholesterol levels potentially predicting a better efficacy.

In summary, the new nutritional score encompassing the serum albumin levels, cholesterol content, and lymphocyte count is more useful in assessing PTB prognosis. After further analyzing the clinical characteristics of the two patient groups (the high and low CONUT score groups), we discovered that the high-scoring group mainly comprised elderly patients, people with underlying illnesses such as diabetes and COPD, and patients in this group may have longer hospital stays. Consequently, more aggressive nutritional measures should be taken to improve the prognosis and shorten the hospitalization duration for patients in the high-scoring group.

In conclusion, a high CONUT score, a low PNI index, and a high NPS score were associated with poor outcomes in PTB patients. These biomarkers are low-cost and widely accessible, making them useful for identifying patients with poor prognoses who may benefit from early nutritional interventions. Further prospective studies are recommended to validate these findings.

Finally, there were some potential limitations to this study. First, we only conducted a nutritional assessment on newly diagnosed patients pre-treatment and did not follow up on their nutritional status. Second, there was limited data on detailed dietary intake during hospitalization as well as other body composition parameters (including body fat or lean body mass). Additionally, this was a retrospective, single-center design study with a relatively small sample size. Moreover, most patients were recruited from Chongqing, and the results may not be extrapolated to other parts of China and the world. Therefore, we intend to conduct additional prospective controlled trials to verify whether nutritional support therapy can improve the prognosis of PTB patients.

## Data Availability

The original contributions presented in the study are included in the article/supplementary material, further inquiries can be directed to the corresponding authors.
